# Risks to Living Kidney Donors – Current Perspectives

**DOI:** 10.31729/jnma.3657

**Published:** 2018-10-31

**Authors:** Badri Man Shrestha

**Affiliations:** 1 Sheffield Kidney institute, Sheffield Teaching Hospitals NHS Trust,, Sheffield, United Kingdom

The first successful living donor kidney transplant (LDKT) was performed on 23rd December 1954 in Boston, USA, where a 23-year-old identical twin donated his kidney to his brother and the donor survived until the age of 81 years with a solitary kidney.^1^ LDKT is the treatment of choice for end-stage renal disease (ESRD), particularly in developing countries, where chronic dialysis is too expensive for most families. LDKT allows for timely transplants and is associated with superior outcomes compared with dialysis or transplants from deceased donors. According to the World Health Organisation, in 2015, 35236 LDKT (41.8% of total kidney transplants) were performed in 102 countries.^2^

Living kidney donors are fully evaluated healthy individuals who voluntarily undergo major surgery of nephrectomy with no physical health benefits to themselves. The perioperative risks, which include haemorrhage, injuries to abdominal viscera, infections, deep vein thrombosis, and chronic wound pain are less serious and are well acceptable. Although rare, the estimated perioperative mortality during organ retrieval from living kidney donors is 0.03% (1 death per 3000 donations).^3^ Kidney donation inevitably leads to reduced renal function and can be associated with increase in proteinuria and rise in blood pressure greater than that can be attributable to normal ageing process. Deterioration of renal function in association with hypertension leading to increased risk of cardiovascular and all-cause mortality.^4, 5^

Living kidney donors often wish to know the long-term outcomes of kidney donation. Due to lack of randomised clinical trials (RCT) comparing the incidence of hypertension, ESRD and death following kidney donation among genetically-related and medically suitable potential donors who proceed to donate or not, current understanding of the long-term outcomes of living kidney donation remains incomplete. Clearly, RCTs of this nature cannot be done for ethical and logistic reasons. However, two large studies published from United States^6^ and Norway^7^ highlight the risks to living donors as compared with equally healthy non-donors.

The study by Muzaale et al, included 96217 US living kidney donors and compared the risk of ESRD among 20024 matched subsets of participants in the Third National Health and Nutritional Examination Survey, who likely would have qualified as living donors. Among the living donors, at a median follow-up of 7.6 years, ESRD developed in 99 (0.1%) donors in mean of 8.6 years after donation. Among the matched nondonors, at a median follow-up of 15 years, ESRD developed in 36 (0.18%) nondonors in a mean of 10.7 years. The estimated risk of ESRD at 15 years was 30.8 per 10000 in kidney donors and 3.9 per 10000 in the matched nondonor counterparts (P<0.001). The relative risk (RR) for ESRD was 8 times higher for the donors and the absolute risk (AR) was 0.3% for the donors.^6^

Mjoen et al, compared the incidence of ESRD, all-cause mortality and cardiovascular mortality in 1901 Norwegian kidney donors with 32621potentially eligible kidney donors, who had median follow-up of 15.1 and 24.9 years, respectively. Nine donors (0.47%) developed ESRD at a median time of 18.7 years from donation, whereas 22 (0.06%) nondonors developed ESRD. The hazard ratio for all-cause mortality and cardiovascular death was 1.30 and 1.40, respectively. The hazard ratio for ESRD in kidney donors was 11.38. The RR for ESRD was 11 times higher for the donors and the AR was 0.47% for the donors.^7^

It is common for young female kidney donors to question about the effect of kidney donation on future pregnancies. A Canadian retrospective cohort study has compared the incidence of gestational hypertension or pre-eclampsia in 85 women kidney donors (131 pregnancies) with 510 healthy nondonors (788 pregnancies) and observed higher incidence of gestational hypertension or pre-eclampsia among kidney donors (15 of 131 pregnancies; 11%) than nondonors (38 of 788 pregnancies; 5%) (Odds ratio 2.5; =0.01).^8^

**Limitations are present in both studies^6, 7^ in terms of follow-up periods, lack of data on family history, mismatch of blood pressure and body mass index and definition of ESRD between the two groups. A recent meta-analysis including 52 studies, comprising of 118426 living kidney donors and 117656 nondonors, showed that donors had higher diastolic BP, lower creatinine clearance, higher risk of ESRD (RR 8.83) and pre-eclampsia in female donors (RR 2.12). However**, the AR for ESRD (0.5 events per 1000 person-years) and pre-eclampsia (5.9 events per 100 pregnancies) were low.^9^

Communication is key to the informed consent process where clinicians play pivotal role in disclosing information on risks relevant to kidney donation that will influence donor's autonomy in making decision consistent with donor's life goals, values and beliefs. The absolute and relative risks should be discussed with the donor at the time of consenting and the need for further research to estimate the risks should be highlighted. The overall risk for ESRD estimated as 8 to 11 times higher than overall risk for nondonors and AR is of less than 0.5% at 15 years and 0.9% lifetime should be emphasised. In clinical practice, it is important to weigh risk profile of each donor in relation to the benefits and risks to the recipient. A young potential donor with lower creatinine clearance will be at higher risk of developing ESRD compared to an elderly donor with similar creatinine clearance.^10^

The impact of evolving techniques of donor nephrectomy ranging from open, laparoscopic, natural orifice transluminal endoscopic to robotic-assisted nephrectomy techniques on the recovery and outcomes should be included in the consenting process. The practice of thorough evaluation during living donor work-up and the long-term follow-up have increased the safety and helped gaining confidence of the donors. Living donors are generous and heroic. It is paramount to provide them the most accurate information regarding risks, especially the risk of ESRD by incorporating the above data into consent process to inform prospective donors fully, which may encourage living kidney donation.

## Conflict of Interest

**None.**
